# Patient safety and sense of security when telemonitoring chronic conditions at home: the views of patients and healthcare professionals - a qualitative study

**DOI:** 10.1186/s12913-023-09428-1

**Published:** 2023-06-06

**Authors:** Mirjam Ekstedt, Espen S. Nordheim, Amanda Hellström, Susanna Strandberg, Heidi Hagerman

**Affiliations:** 1 Faculty of Health and Life Sciences, Department of Health and Caring Sciences, Linnaeus University, Universitetsplatsen 1, Kalmar/Växjö, 392 31 Sweden; 2 Department of Learning Informatics Management and Ethics, Karolinska Institutet, Stockholm, Sweden; 3 Norwegian Centre for E-health Research, University Hospital of North Norway, Tromsø, Norway

**Keywords:** Chronic disease, Cross-sectional study, Health literacy, Home healthcare, Patient safety, Primary healthcare, Security, Self-management, Telemedicine

## Abstract

**Background:**

Chronic diseases are increasing worldwide, and the complexity of disease management is putting new demands on safe healthcare. Telemonitoring technology has the potential to improve self-care management with the support of healthcare professionals for people with chronic diseases living at home. Patient safety threats related to telemonitoring and how they may affect patients’ and healthcare professionals’ sense of security need attention. This study aimed to explore patients’ and healthcare professionals’ experiences of safety and sense of security when using telemonitoring of chronic conditions at home.

**Methods:**

Semi-structured interviews were conducted with twenty patients and nine healthcare professionals (nurses and physicians), recruited from four primary healthcare centers and one medical department in a region in southern Sweden using telemonitoring service for chronic conditions in home healthcare.

**Results:**

The main theme was that experiences of safety and a sense of security were intertwined and relied on patients´ and healthcare professionals´ mutual engagement in telemonitoring and managing symptoms together. Telemonitoring was perceived to increase symptom awareness and promote early detection of deterioration promoting patient safety. A sense of security emerged through *having someone keeping track of symptoms* and comprised aspects of availability, shared responsibility, technical confidence, and empowering patients in self-management. The *meeting with technology changed healthcare professionals’ work processes, and patients’ daily routines*, creating patient safety risks if combined with low health- and digital literacy and a naïve reliance on technology. *Empowering patients’ self-management ability* and improving shared understanding of the patient’s health status and symptom management were prerequisites for safe care *and* the patient´s sense of security.

**Conclusions:**

Telemonitoring chronic conditions in the homecare context can promote a sense of security when care is co-created in a mutual understanding and responsibility. Attentiveness to the patient’s health literacy, symptom management, and health-related safety behavior when using eHealth technology may enlighten and mitigate latent patient safety risks. A systems approach indicates that patient safety risks related to telemonitoring are not only associated with the patient’s and healthcare professionals functioning and behavior or the human-technology interaction. Mitigating patient safety risks are likely also dependent on the complex management of home health and social care service.

## Introduction

Healthcare systems worldwide face a growing challenge in dealing with the increasing prevalence of long-term diseases. Chronic diseases and multimorbidity have strong associations with age, a progressively reduced functional status, and increased use of inpatient and ambulatory healthcare [1]. The complexity of disease management increases the need for safe and efficient provision of the comprehensive care that people living with chronic conditions need [2]. A key is to empower patients in self-management, through personalized information and support [3]. eHealth technology, in particular telehealth, promises benefits through improving self-management of chronic conditions and providing timely access to high-quality care at a distance [4, 5]. Self-management by use of telemonitoring [6] commonly includes opportunities for patients to receive regular feedback and explanations of symptoms, enabling them to take action if their health status deteriorates [4]. It also has the potential to enhance patients’ sense of security through support, monitoring, reminders, and facilitating trusting and reliable relationships with healthcare professionals over time [7, 8]. The introduction of various telehealth technologies in patients’ homes has thus created possibilities to help people with chronic conditions remain at home, aging in place [8].

Although telemonitoring may facilitate disease control and self-management of chronic conditions, it can also introduce new patient safety risks, with unanticipated consequences [9, 10]. Implementation of new technology in daily practice is challenging, as the scale of change that this entails is unprecedented [11] and it involves a range of stakeholders with differing backgrounds and interests (i.e., healthcare professionals, technicians, developers, patients, and family members). Studies show shifting results regarding outcomes of telemonitoring [4]. Some reviews report reduced healthcare consumption and costs and improved quality of life [12], reduced mortality and improved self-management of the disease [13] while others do not show any differences when comparing with self-management support from usual care [14]. Research involving vulnerable groups is even less cohesive [15]. Studies of patients with heart failure have shown that their ability to perform self-care may be overestimated and that low self-care ability is associated with higher mortality risk [16]. When using telemonitoring for disease control and self-management of chronic conditions, health literacy, and digital literacy are important factors that affect the outcomes of the care. Health literacy is defined by Berkman et al. [17] as *“The degree to which individuals can obtain, process, understand, and communicate about health-related information needed to make informed health decisions”.* UNESCO [18] defines digital literacy as “*The ability to access, manage, understand, integrate, communicate, evaluate and create information safely and appropriately through digital technologies*”. Low levels of health literacy and digital literacy may be a discriminating factor resulting in exclusion of vulnerable groups and introducing risks that impact the outcome of telemonitoring in chronic conditions [15]. In a systematic review, Parker et al. [15] state that low levels of health literacy can affect the patient’s motivation to act, further emphasizing the importance of supporting health and digital literacy in the context of telemonitoring for chronic conditions.

Patients who can benefit from self-monitoring at home need support to manage telemonitoring devices and integrate them into their daily lives. They also need support from healthcare professionals to interpret their own health data and take action to revise planned treatment and understand how to manage symptoms [19]. While it is essential that patients feel secure when using technology, it is likewise important that healthcare professionals feel confident when using the systems. Even in countries with high technology usage, professionals are often unfamiliar with the use of noninvasive telemonitoring in daily follow-up care [20]. Despite the growing evidence on the impact that use of telemonitoring devices has on disease control and patients’ self-management ability [13], there is a need to monitor developments carefully and mitigate risks where they arise. Threats to patient safety stem largely from the conditions of healthcare work within complex socio-technical systems. Traditionally, patient safety research is focused on preventing and reducing risks, errors, and harm to patients in healthcare [21]. The incidence of adverse events in home healthcare is estimated to occur in one third of patients, and most are deemed preventable [22, 23]. However, performing safety work for older people living at home is quite different from hospital care [24]. In the home environment, a holistic approach is needed that along with the healthcare system´s structures and processes of care also include the patient´s functioning and behavior as well as the social and physical environment. Lette et al. [25] states that professionals would benefit to have an interdisciplinary and multidimensional way of understanding when addressing older people´s safety. Lette et al. [25], has based on Lau et al.’s [26] framework for health-related safety, categorized five domains of risks that have impact on patient safety in home environment: patient functioning and behavior, social environment, physical environment, and health, and social care management. Risks and problems are likely also associated with a patient’s confidence and sense of security in performing self-care linked with a trustworthy relationship with healthcare. Sense of security is defined by Petersson and Blomqvist [7] as “*an intrinsic state based on faith and trust in oneself and others*”. Healthcare professionals play a crucial role in building trustworthy relationships and in this way supporting patients’ sense of security. In addition, external factors such as being part of a community, being familiar with things, and using various kinds of aids can strengthen the sense of security [7]. From this broader perspective, the probability that a person with chronic conditions will continue to live an independent life and age in place is likely related to both patient safety [25] and a sense of security [27].

Given the global trend towards large-scale implementation of telehealth and self-monitoring of chronic conditions, there is a need for all stakeholders to know whether and under what circumstances the introduction of telemonitoring in patient homes can contribute to the safety of care and a patient’s sense of security. This study aimed to provide a deeper understanding of patients’ and healthcare professionals’ experiences regarding patient safety and a sense of security when telemonitoring chronic conditions in home healthcare.

## Methods

### Study design and setting

A qualitative, inductive approach using semi-structured interviews was considered appropriate to provide a comprehensive understanding of the end-user’s experiences of latent and manifest threats to patient safety and sense of security with telemonitoring chronic conditions in the home healthcare context [28]. This study was embedded in a pilot project conducted in a region in southern Sweden, aimed at implementing a telehealth application and devices for telemonitoring of chronic conditions in primary care. The region where the pilot was launched has about 250,000 inhabitants spread across a large geographic area, with among the highest proportions of older adults in the country.

Four primary healthcare centers and one medical department at a district hospital were selected by the region to pilot the telemonitoring service for chronic conditions in primary care. The purposes of the pilot were to test if remote health control (telemonitoring) of chronic diseases could increase safety, security, patient participation, and continuity in care, and in the long term reduce the number of emergency visits in patients with chronic conditions. How the value formation through eHealth interactions is created from the managers’ and healthcare professionals’ perspectives are presented elsewhere [29]. During six months, a total of 65 patients with heart failure or hypertension had access to the telehealth application for tablets and smartphones. Patients needed to have sufficient cognitive and functional ability to use devices for video consultation and self-monitoring of bodyweight, blood pressure, heart rate, oxygen saturation, body temperature, blood glucose level, and other vital signs and symptoms that reflect their health status. Nurses without or together with service technicians introduced the patients and started up telehealth applications on a tablet or a smartphone. Patients, sometimes assisted by family carers, used the devices in their home environment to monitor diagnosis-specific values in accordance with an individual plan set up with the physician. The data generated were transferred over a secure internet connection to primary healthcare centers or the medical department at a district hospital. Nurses at the care departments performed daily monitoring and registered health data in each patient’s electronic health record regularly, Monday to Friday. Nurses gave self-care guidance to the patients via chat or video in the telehealth application. In case of deviating values and urgent matters, nurses consulted a physician and contacted the affected patient by telephone, made a home visit, or made an appointment for the patient at the care department. Only nurses had contact with the patients through the application. The organizational preconditions in terms of time to work with the application differed. Some nurses had time scheduled to work with the application, whereas others did not.

### Participants and data collection

All healthcare professionals (seven nurses and two physicians) who worked with the project at the five units were informed about the study and asked by the last author (HH) to participate in an interview. All agreed and gave written consent. The nurses at each unit gave information to their patients about the study. A purposive sample of four to five patients per unit was employed to select the participants that could provide rich information about the research question and achieve variation in sex, age, and chronic condition [28] (Table 1). Twenty-three patients that were interested in participating were contacted by SS. Twenty gave written consent and chose to participate. Semi-structured interviews were collected between March and May 2020. HH performed the interviews with healthcare professionals and SS conducted the interviews with patients. Both are Registered Nurses and are experienced in qualitative interviewing. Due to the COVID-19 pandemic, all interviews were performed via Skype, Zoom, or telephone. The interviews were guided by an interview protocol with open-ended questions. The protocol was developed and discussed within the research team and covered themes about patients’ and professionals’ experiences of safety risks and feelings of security, difficulties and enablers regarding digital communication and monitoring, patients’ social and physical home environment, and professionals’ working environment. The interviews lasted between 30 and 60 min and were digitally recorded. No repeated interviews were carried out.

**Table 1:** Overview of the participants

**Patients**	**n = 20**
Age in years, Md (max/min)	72.5 (50–88)
Sex, female	9
Diagnosis	
*Heart failure*	13
*Hypertonia*	7
*Diabetes*	0
**Healthcare professionals**	**n = 9**
Age in years, Md (max/min)	52 (39–58)
Sex, female	9
Profession	
*Registered nurse*	7
*Physician*	2
Years in profession, Md (min/max)	13 (6–38)
Years in workplace (min/max)	6 (1–30)

### Data analysis

The interviews were transcribed verbatim and analyzed using an inductive content analysis approach [30]. The author (ESN) who is a research assistant and has a master’s in sociology took the lead in coding data with ME, HH, AH, and SS, all registered nurses and experienced in qualitative methods, as discussion partners. All transcripts were coded using NVivo (QSR International) to promote handling of large dataset. The first step in the analysis was reading through the interviews to get a sense of the whole and grow familiar with the text. Then, meaning units with content related to the study aim were extracted and labelled with codes, with references to the informant’s raw data. In the next step, the codes were sorted into one main theme and four subthemes by going back and forth between codes and raw data and comparing differences and similarities in the participants’ statements. The main theme comprised the latent content of the four subthemes, illuminating the underlying meaning of the shared experience of safety and security among patients and healthcare professionals when using telemonitoring in home healthcare [31]. The four subthemes corresponded to a diversity of experiences related to safety and a sense of security using telehealth application and telemonitoring devices. The whole research team was involved in the sorting and abstraction of codes into subthemes and a main them, to enhance trustworthiness by continuing the analysis until consensus was reached [28, 31]. The interviews and analysis were performed in Swedish. The quotations are translated from Swedish to English language by a professional editor.

## Findings

The main theme, *experiences of safety and a sense of security are intertwined and rely on mutual engagement in digital monitoring*, captures that both patients and healthcare professionals experienced that self-monitoring in a patient’s home created commitment and new opportunities in managing symptoms together. Patients could learn to live with their chronic health conditions at home and both patients and professionals described increased safety awareness and a sense of security through the management of care they performed together.

The first of the four subthemes that described experiences related to safety and security was *increased availability enabled through telemonitoring*. The mutual responsibility in monitoring clinical values promoted a sense of security among both patients and healthcare professionals. It was perceived as helpful *having someone keeping track of symptoms*. The participants described that *the meeting with technology changed work and daily routines*, creating risks if combined with low digital literacy and a naïve understanding of how technology functions. Finally, it was found that *empowering patients’ ability in self-management* improved shared understanding of the patient’s health status and symptom management, which was seen as a prerequisite for safe care performance and a sense of security. The four subthemes are further described below.

### Increased availability enabled through telemonitoring

The patients and healthcare professionals described that telemonitoring reduced the distance between them and made them more available to each other. The nurses said that the patients got a fast track into the healthcare system, and patients agreed that there was a direct line to the healthcare professionals.

“The staff is just a few keyboard strokes away – that feels good.” [Patient 014].

The availability of the telehealth application and the routines related to self-monitoring made patients feel seen by the healthcare system, which was a new experience for some patients and contributed to a sense of security. Patients also perceived care as safe by the availability to interact with the same physician or nurse who was familiar with their health history. The nurses in turn saw it as securing for patients to have regular care contact, whether through telemonitoring or not. Both patients and healthcare professionals said that conversations between them expanded from being merely health-related to including social aspects and other areas of life, which created a bond of trust and security. This in turn reduced the risk of misunderstandings and something going wrong. However, not everyone felt that they bonded, and some concerns about nobody would be there were raised.

“… I don’t know, someone wrote to me once and sent an attachment, and then I wrote back, and I didn’t get a response. And then it feels like … oh, like, isn’t there anyone at the other end? And then I was like – ‘what!?’ And calling them, that doesn’t seem to work either.” [Patient 003].

Some healthcare professionals described challenges in working with the application due to time constraints. They described availability as an aspect that could create tension in the workplace, as they got messages throughout the day, and answering them might be deemed a misuse of time. Some nurses said it was hard to end conversations with patients, especially when they involved complex and emotionally charged topics.

### Having someone keep track of symptoms

Many patients described increased confidence from knowing that the healthcare professionals checked their health data and would contact them if the values deviated.

“Yeah, that they keep an eye on me. Every week, they can see what my values have been during the entire week, and can see what they were on Monday and they can see what they’ve been every day. So that creates a sense of security.” [Patient 009].

Even during acute deteriorations, the healthcare professionals were able to keep the patient confident through heightened monitoring and management of the patient’s symptoms via telemonitoring. Another aspect that both patients and professionals described as contributing significantly to safety was the monitoring over an extended time period. The frequent measurements and long-term control increased symptom awareness with improved quality of care, especially for patients who would typically get a health check-up annually or biannually. The healthcare professionals saw long-term monitoring as a way to detect early signs of deterioration in a patient’s health. Furthermore, they described that the patients were more closely looked after and monitored than patients who did not have access to self-monitoring using telemonitoring. Some patients expressed trust arising from someone checking their values every day, even on weekends. However, the nurses described that this was not the case as they only checked the values during office hours Monday through Friday.

“… and we have seen in one patient, who we’ve given an increased dose of blood pressure medication. He hadn’t felt anything…hadn’t had any symptoms, but we’d seen it through the home monitoring and been able to get the blood pressure down, pretty well anyway. That was a patient who’d had a stroke in the past, so it was important.” [Healthcare professional 3].

### The meeting with technology changed work and daily routines

Both patients and healthcare professionals described the meeting with technology as a new and ambivalent experience affecting their everyday routines. Some healthcare professionals said they were initially skeptical about using eHealth technology at work, but changed their minds when they became familiar with the telehealth application.

“In the past, I’ve seen eHealth as something kind of negative and I’ve thought a lot about how the personal interaction disappears and that you don’t meet face to face and that whole aspect, but I’ve had to change my views. I think this works really well.” [Healthcare professional 5].

Patients described similar experiences, that they were positively surprised at how well they could manage the technology and integrate the new routines into their lives. However, both patients and healthcare professionals mentioned some technical problems that affected their experience negatively and might jeopardize safety. Some patients were uncertain about correct use of the devices (step counter, webcam, blood pressure monitor), if the devices were connected correctly and whether the data had been registered. The healthcare professionals also described their main concern as related to if the measured health data were registered correctly.

“Yeah, well sometimes you receive a bodyweight of 4 kilos … or a body temperature of 26 degrees, and then we know that this … this isn’t right.” [Healthcare professional 2].

Many healthcare professionals mentioned that the telemonitoring had features that mitigated risks and adverse outcomes. For example, an alert was sent if a patient’s values were above or below specific values, which was helpful for detecting deteriorations at an early stage. Another appreciated functionality was the possibility of performing video meetings with patients. Such meetings allowed the healthcare professionals to visually examine the patient’s condition and detect deterioration, for example if a patient with heart failure was cyanotic and had blue lips.

### Empowering patients’ ability in self-management

Patients described that self-monitoring using telemonitoring gave them an overview of how their health data varied over an extended period of time, giving them an opportunity to understand how their body worked. Monitoring disease-specific values was perceived as having control over the disease, which created a sense of security.

“Yeah, but you see it, you see the pressure and all that and I think that’s the thing, the pressure, that it’s been pretty good now. It’s staying … it’s fairly steady and staying within the reference values. So that feels safe, that must be good, I think. So that … yeah, but I feel safe because of that.” [Patient 004].

However, not all patients felt secure by measuring their values regularly. One patient said it was stressful to get a daily reminder of his condition, especially when it deteriorated. Some patients described that the monitored health data created a sense of security only if they could process and understand them. The healthcare professionals for their part, experienced self-monitoring as a useful tool for educating the patients on how to prevent or mitigate deteriorations and how to take responsibility for their own health.

“I think of it like this, newly diagnosed patients who need to learn more about their disease, that’s where I think you have the greatest gains (with home monitoring).” [Healthcare professional 1].

## Discussion

**Fig. 1:**
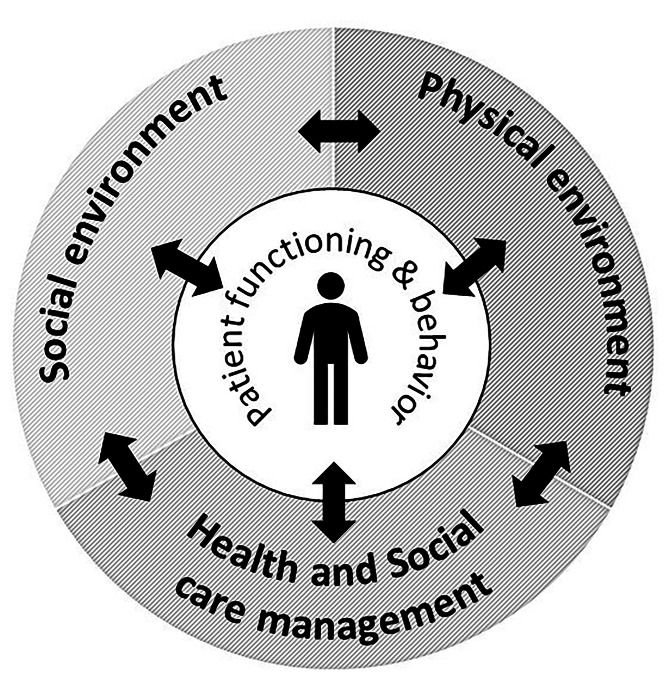
Domains of safety risks for people living at home adapted from Lette et al. [25]

This study provides insights into perceptions of safety and a sense of security when patients with chronic conditions use telemonitoring for disease control and self-management support at home. The main findings suggest that safety and a sense of security are closely intertwined and rely on mutual engagement from both patients and healthcare professionals. Self-monitoring required firm commitment on the part of patients and enhanced their understanding of symptom management. The results will be discussed in relation to Lette et al´s [25] five risk domains (i.e., client/patient functioning and health behavior, social and physical environment, and healthcare and social care management), (Fig. 1) that are likely to impact on patient safety and sense of security [7] in home healthcare when self-monitoring using telemonitoring is introduced [25, 26].

### Patient functioning and behavior

This study suggests that the capacity for a telemonitoring system to operate safely lies in the possibility of achieving a common foundation for understanding a patient’s symptoms and health behaviors. Taking a holistic perspective on patient safety means viewing the patient as an integrated entity where the patient’s functioning and behavior are highly interrelated as illustrated in Fig. 1. This implies for example that the patient’s management of symptom burden affects and is affected by the person´s health behavior. Self-monitoring of health data provides instant access to information that facilitates actions to promote healthy behavior in daily life [5]. Experiences of safety were related to a patient’s behaviors in processing the health data and adapting in accordance therewith. The monitoring of health data improved patients’ sense of security, as they knew that the healthcare professionals were “watching them” from a distance. However, blindly trusting that “someone else” was in control might create a false sense of security and could introduce a risk, as the nurses did not monitor data on weekends. Patients needed to have a certain measure of health literacy to understand their symptoms and actively seek care in case of acute complaints [5]. Healthcare professionals described it as meaningful to educate patients about their health and symptoms and guide them in adjusting treatment or self-care behaviors. The findings thus indicated that a sense of security would emerge from a mutual understanding of a patient’s health and symptom management and an improved awareness of health-related safety behavior [25, 26]. Like other studies, this points to the importance of the co-production of common goals where the patient’s value is central [29, 32].

Telemonitoring offered opportunities for two-way communication between patients and healthcare professionals enabling information exchange – both synchronously, using the chat function, and asynchronously, via telemonitoring of self-assessed health data. Healthcare professionals highlighted the potential of building stronger relationships by interacting with their patients and the potential of early detection of deterioration and rapid adjustment of treatment. Patients, in turn, highlighted that easy access to healthcare was essential for their sense of security at home. This finding is in line with those of another study showing that accessibility to the healthcare center through telehealth improved the possibility for communication and reciprocal relationships, which is important for a shared understanding between patients and healthcare professionals and contributes to the sense of security [3, 19].

### Social and physical environment

Limitations of the technology or the physical home environment, such as an unstable internet connection, have been shown to increase risks for harm [19]. As described by Sitting et al. [33] there is a need for all stakeholders to recognize that flawless software has never been developed, and that safety-critical situations and social and environmental risks must be carefully monitored in the implementation of telehealth. This study provides insights into how uncertainty regarding the use of telemonitoring and occasionally insufficient quality of telemonitoring devices contributes to incorrect values. Safety risks may also be related to patients’ digital literacy and cognitive and physical functioning. However, health literacy and digital literacy are rarely investigated in studies on telehealth tools in vulnerable patients [15]. Social support from a trusted healthcare professional or next-of-kin might mitigate technical problems when they arise. In contrast, functional declines when living alone, at a distance from support, can be expected to exacerbate such problems. As others have discussed [7], consistent relationships over time, where healthcare professionals get to know the patients and what matters to them, may improve patients’ sense of security and mitigate safety risks.

An aspect that both healthcare professionals and patients appreciated was that monitoring was long term, as it not only increased disease control and a sense of security, but also promoted trusting relationships and patient competence for self-care [27]. The extent and intensity of self-monitoring are worth considering, as the need for support varies greatly between individual patients with chronic conditions, depending on background, illness severity, functionality, social support, home environment, et cetera.

### Health and social care management

Aligning self-monitoring with healthcare professionals’ workflow and priorities is equally important, as their endorsement and engagement are key to safe management of a telemonitoring service and patient self-care support. In line with results from other studies, participants in the current study expressed ambivalent feelings regarding the accessibility through telemonitoring [34]. Healthcare professionals described an increased workload, highlighting that heightened awareness of patients’ situations can increase ethical stress among staff [35, 36]. However, the importance of organizational preconditions in terms of time dedicated to working with telemonitoring data became apparent.

This study underlines the organizational responsibilities of providing personalized, adapted support to both patients and healthcare professionals when introducing telemonitoring into a real-life setting. The results indicate that the implementation of self-monitoring devices in everyday life for patients requires shared responsibility and a socio-technical approach, i.e., a focus on the people, processes, environment, and technology involved [33, 37]. Vulnerable patients with low social support, poor health, and digital literacy, or physical or cognitive impairments are likely the ones who would benefit most from telemonitoring, but would also be at the highest risk through use of such a service [15]. It might be beneficial to consider telemonitoring as a supplement to regular practices, to empower people with chronic conditions to age in place, feeling safe and secure. Given the complexity and safety-critical nature of home care of people with chronic diseases, a dedicated coordinator role that translates telemonitoring services into chronic disease management pathways is suggested. If appropriately adapted to each patient, telemonitoring has the potential to contribute to safety and security at home from a health-related, holistic perspective [26] – something that patients have a legal right to [38].

### Limitations

It must be noted that the findings in this study are based upon the experiences of healthcare staff and patients using the telemonitoring devices, and not objective measures of risks as such. This could be considered a weakness, as feeling safe does not mean the same as not being exposed to patient safety risks. On the other hand, this study provides knowledge on user needs, real-life experiences, and user characteristics that may contribute to a better understanding of direct and latent safety risks appearing in the home care context.

Due to the COVID-19 pandemic, interviews had to be held by telephone or as video meetings. This was not according to plan but is not considered to have affected data collection negatively, which is in line with Archibald et al. [39]. The authors (HH and SS) who conducted the interviews did not perform the primary analysis, meaning that the first impression of the data was text-based. There may also be a strength in the fact that ESN (who conducted the first analysis) being male and with a different professional background than the other four authors (all females and registered nurses) went into the analysis with lesser preunderstanding and fresh eyes. However, as all authors were involved throughout the subsequent steps of the analysis, which ensured the credibility of the analysis and that the voices did not get lost [28]. One limitation might be that patients were selected by the nurses conducting the pilot project. Patients might have been chosen based on their willingness to use digital technology. There might also have been patients who were excluded due to ethnicity, a lack of technical or digital skills, or being too ill. In addition, the interviews did not include questions about integrity and transfer of personal data. The sample size was considered sufficient due to homogeneity of the study participants; all had experience of the research topic and the interviews provided rich data. When data coding did not provide more variation, saturation was considered to have been reached [28]. We did not use member checks as the value of participants checking if the findings are true to their experience are disputed [28]. An audit trail of quotations from the interviews is left for the reader’s judgment if the results are transferable to other contexts [31].

## Conclusions

Patients’ and healthcare professionals’ experiences regarding safety and a sense of security when telemonitoring is used for chronic disease control and self-care support in home healthcare are interrelated and dependent on mutual engagement. Experiences of patient safety and a sense of security when using telemonitoring at home are related to a patient’s functioning and behaviors in processing the health data and adapting accordingly. Healthcare professionals’ attentiveness to the patient’s health literacy, symptom management, and health-related safety behavior when using eHealth technology may mitigate latent patient safety risks. Self-monitoring using telemonitoring devices creates new opportunities for people with chronic conditions to live independently and age in place if the safety aspects of home care are fully considered. The five risk domains, adapted from the framework by Lette at al, [25] may be useful for policymakers and managers for adopting a system-wide perspective on potential patient safety threats in the home healthcare setting that account for the patient’s physical and cognitive functioning and health behavior and the social and physical home environment. (Fig. 1). This study also highlights that patient safety risks when telemonitoring chronic conditions in patients’ homes are not only related to the people, tasks, or technology involved but are likely also embedded in the complex management of home health and social care service.

## Data Availability

The interview data that were collected and analyzed in this manuscript are not publicly available due to participants not having consented to public availability. However all the datasets and aggregated data in Swedish are available from the corresponding author on reasonable request.
